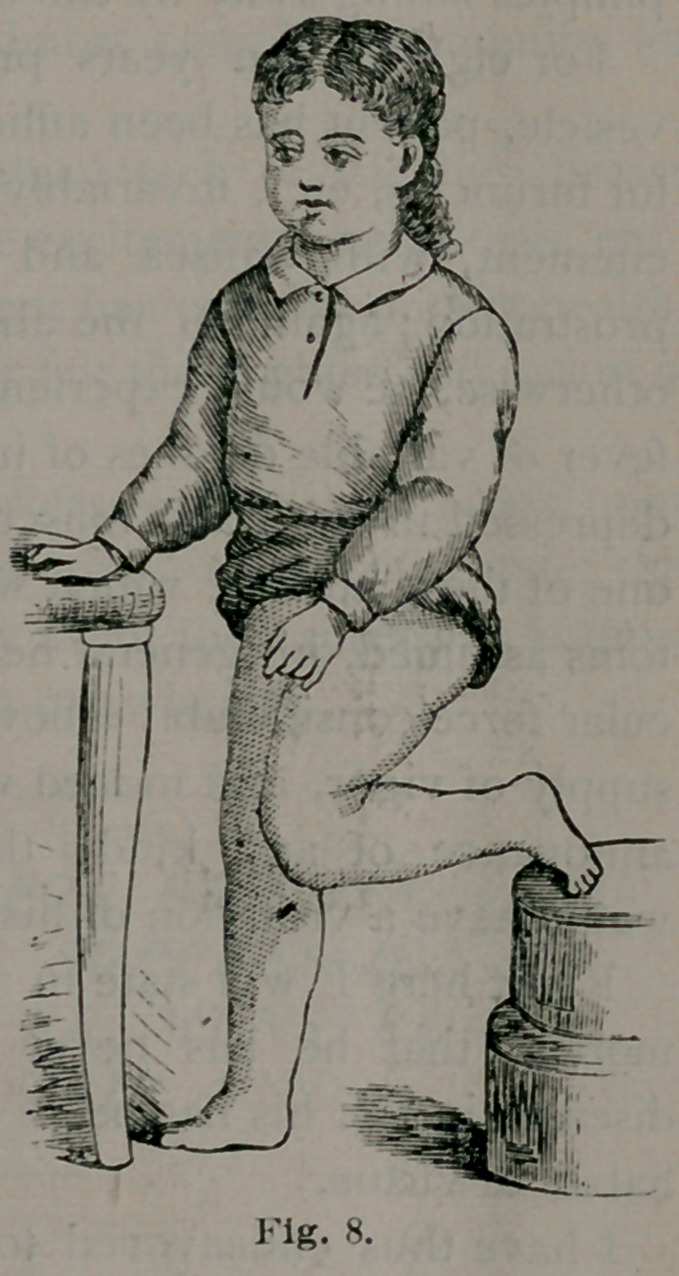# Brooklyn Anatomical Club

**Published:** 1879-07-20

**Authors:** Geo. R. Fowler


### *BROOKLYN ANATOMICAL AND SURGICAL CLUB.*

REPORT, BY GEO. R. FOWLER, M. D.

Congenital deformities of the knee-joint are very rare. The patella is occasionally found to be dislocated either to the inner or outer side of the leg at birth, but other than this, no departure from the normal shape of the limb at the knee-joint seems to occur congenitally.

Genu-valgum is one of the most common deformities met with. It is seldom acquired during middle life or old age, but generally occurs during the period extending from infancy to adolescence.

The stages of development of this deformity are believed to be as follows :

1st. Disproportionately powerful contraction of the biceps flexor cruris muscle.

2d. Relaxation of the internal lateral and posterior crucial ligaments.

3d. An arrest of growth of the external and increased growth of the internal condyle of the femur.

4th. Oblique outward rotation of the tibia.

When the deformity occurs during infancy, it is generally during the period of most difficult dentition, after the child is a year old, and when abnormal states of general nutrition more commonly obtain.

The cases which occur later in life are met with most frequently in the persons of those who are apprenticed at an early age to some trade requiring constant standing. Of these, the greater number will be found among bakers and cabinet-makers; and indeed, so common is this deformity in Germany among the first-named, that it is there known as "baker's leg." Among bakers it occurs almost invariably in those whose duty is to "scale" or weigh the dough after it is rolled out in loaves for baking. These persons stand with their right limb thrown slightly forward, the knee flexed, and the foot somewhat in the position of splay foot. The biceps muscle of the right limb is rigidly contracted, in order to assist the workman in maintaining his equilibrium as he receives the dough from the right-hand side of the scales, weighs it, and then rotates his trunk upon his lower extremities to pass it along to the oven. Any one imitating this position and motion will be at once struck by the rigid condition of the outer hamstring of the right limb, or the one thrown forward, as compared with the relaxed inner hamstring of the same limb.

The effect of this disproportionately powerful contraction of the biceps is to put upon the stretch the internal lateral and posterior crucial ligaments. These slowly yield to the constant traction until they become permanently relaxed, and the articulation, instead of having simply an antero-posterior motion, the only one it possesses in a normal condition, gains lateral movement.

This lateral movement tends to a deviation in the normal shape of the limb when the body is in the erect position. The vertical column which the healthy limb represents has its extremities and fixed points respectively at the ankle and acetabulum, and supports the weight of the trunk in the line of its axis. When relaxation of the internal lateral and posterior crucial ligaments occurs the femur maintains its original and perfect position; but the feet are removed to a greater distance from each other, and the weight of the body is received upon a broken line, the axis of the femur meeting that of the tibia at the knee-joint, and forming the apex of a triangle, the base of which is represented by a line drawn from the centre of the acetabulum to a point midway between the two malleoli. As will be readily seen, this deviation from the normal shape of the limb must necessarily result in an increased pressure upon the external condyle of the femur, and an almost entire relief from the pressure of the internal condyle. The external condyle, in this abnormal condition, bearing, as it does, half of the entire weight of the trunk, is arrested in its growth, and the internal condyle becomes lengthened. The limb, when flexed, maintains its normal position and relations; partially extended, the tibia rotates obliquely outwards, and in full extension the lengthened internal condyle fills up the space which would otherwise exist between it and the head of the tibia, and the normal axis of the limb, as a whole, is destroyed.

In the treatment of this deformity nothing can be gained by waiting for Nature to right the limb. Spontaneous recovery never takes place, and mechanical treatment is frequently of no avail, unless conjoined with division of the tendon of the biceps. As long as there is lateral movement to the knee-joint something may be gained by attempting to restore the normal axis of the limb by mechanical means, together with tenotomy of the external hamstring. But when no lateral movement can be demonstrated to exist, other operative measures become necessary.

The earlier attempts to correct this deformity by operative procedure other than tenotomy consisted of removal of a wedge-shaped piece of the head of the tibia by what was known as Meyer's operation. This has been most emphatically and justly condemned as based upon incorrect and unsound principles. Knock-knee is not dependent in any way upon cuivature or other alteration in the size and shape of the tibia.

After the introduction of Lister's methods of antiseptic surgery, operations involving the opening of joints became more frequently resorted to, and attempts to correct genu-valgum by sawing off and removing the elongated internal condyle were made.

This operation (Anandale's), although based upon a knowledge of the anatomy and pathology of the deformity, did not become very popular with surgeons, for the reason that it almost invariably resulted in a complete and incurable bony anchylosis of the knee-joint, and was finally abandoned.

In 1876 Dr. Ogston, of Aberdeen, Scotland, proposed as a means of relief of this deformity an operation consisting of the subcutaneous division and fracture of the internal condyle of the femur, and a forcible straightening of the limb, thereby restoring the normal proportion in the length of the two condyles without removing any part of the articular surface. Subsequently, in *The Edinburg Medical Journal* for March, 1877, he reported a case so operated upon in May, 1876, under the antiseptic spray of Lister, in which a perfect cure was accomplished. Mr. George W. Callender, of London, as well as other English surgeons, has since repeated the operation of Ogston—without the antiseptic spray, however—with the same excellent results.

This operation, as performed by myself, is as follows: The limb being strongly flexed and rotated outwards, an Adams tenotomy knife is entered about two inches and a half above the tip of the internal condyle of the femur, and in the middle line of the inner aspect of the thigh; with its edge directed towards the bone, the knife is pushed onwards, until its point can be felt to have reached the groove between the condyles. With the knee strongly flexed and the patella drawn outwards (if it be not already dislocated), it is not difficult, through the tightly-drawn anterior coverings of the joint, to feel the exact location of the knife after it has entered the cavity of the joint. A saw such as Mr. Adams uses in performing subcutaneous osteotomy of the femur is passed along the same route, the flat side of the knife acting as a guide. The latter is then withdrawn. The bone is sawn in the direction of the dotted line, Fig. 1, by short strokes directly backwards, and when

Fig. 1.

Fig. 2.

it is judged to be nearly divided, the limb is extended and forcibly straightened, the inner condyle being fractured and forced upwards in a position to bring its articulating surface upon a level with that of the external condyle as shown in Fig. 2. This being accomplished, the limb is retained in position by some fixed dressing. About the fourteenth day passive motion is commenced.

The two following cases, occurring in my own practice, were submitted to this operation with the most gratifying results:

*Case* 1. Joseph Redman, aged 19: a baker by occupation; born in Germany; one year in this country. About three years ago, and shortly after being apprenticed to his present trade, he noticed the deformity. It steadily increased, and when he came under my observation it was in the condition shown in Fig. 3, from a photograph. On

Fig. 3.

Fig. 4.

Nov. 26th, 1878, I performed Ogston's operation as above described, under carbolic spray, in the presence of Drs. Pilcher, Jewett, Hunt, Elmendorf and King. The anæsthetic used was ether. After the operation the wound was dressed with a single layer of antiseptic marine lint and covered by Macintosh. The limb was then put up in plaster of Paris, supported by a short thigh splint. No reaction occurred, and the patient remained absolutely free from all pain and discomfort.

On the fourteenth day I removed the dressings for the first time, and found the wound healed perfectly. Passive motion was then commenced, and in less than three weeks after the operation the patient walked about the room.

Fig. 4 shows the condition of the limb at this time, and Fig. 5 the amount of flexion he can comfortably make at the end of three months.

Fig. 5.

*Case* 2. Annie Behrman, aged 2 years and 10 months, of German parents; a healthy child in other respects. At nine months she began to walk, but it was not until six months afterward that the knock-knee was observed. The parents applied to an instrument maker, who made for her a long brace with a joint at the knee and elastic bands. This was worn for four months, and abandoned, no benefit being derived from the treatment. For several months nothing was done for the child. She was then brought to Mr. Leyh, a skillful maker of surgical mechanical appliances in the Eastern District, who sent her to me for operative treatment.

Fig. 6 (on next page), from a photograph, shows her condition at this time. The "out-knee" of the other limb and a lateral curvature of the spine are secondary effects of the genu-valgum.

On April 5th, 1879, I performed Ogston's operation upon this child under the carbolized spray; present, Drs. Pilcher, Figueira, Elmendorf, King and Rogers. I had caused to be made a small Adams saw especially for this case. I sawed the internal condyle completely through. Upon trial it was found that the deformity, although considerably lessened, could not be completely reduced. I then resorted to subcutaneous division of the tendon of the biceps flexor cruris, after which the limb was easily straightened. The wounds were dressed with antiseptic marine lint and carbolized oil-silk, and the limb encased in a paraffine splint. The child was then laid in Prof. F. H. Hamilton's double splint for fracture of the thigh occurring in children, and the limb operated upon securely bandaged to the apparatus. The other limb was also secured to the splint on that side.

The reaction resulting from so severe an operation upon so young a child was surprisingly slight. The temperature never arose above 100° Fahr., and, after the first night, no pain was complained of. On the fourteenth day I removed the dressing and found the wound entirely healed. The limb was perfectly straight, as shown in Fig. 7, and I at once flexed it to a right angle. For the first few days the limb was supported by placing the child in the double splint after each act of passive motion, and cold water dressings applied. Fig. 8 shows the power of flexing the limb now possessed by the child.

Eig. 6. Fig. 7. Fig. 8.

These two cases are believed to be the first operated upon by this method in this country.

## Figures and Tables

**Fig. 1. f1:**
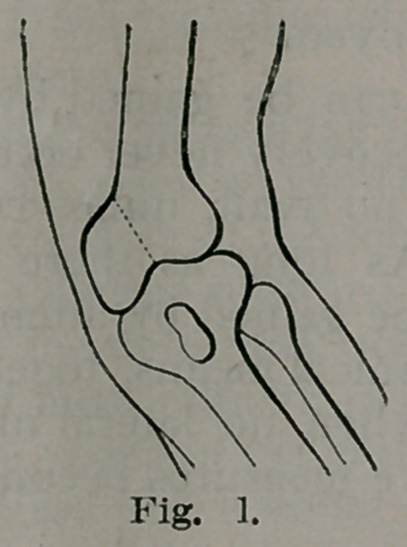


**Fig. 2. f2:**
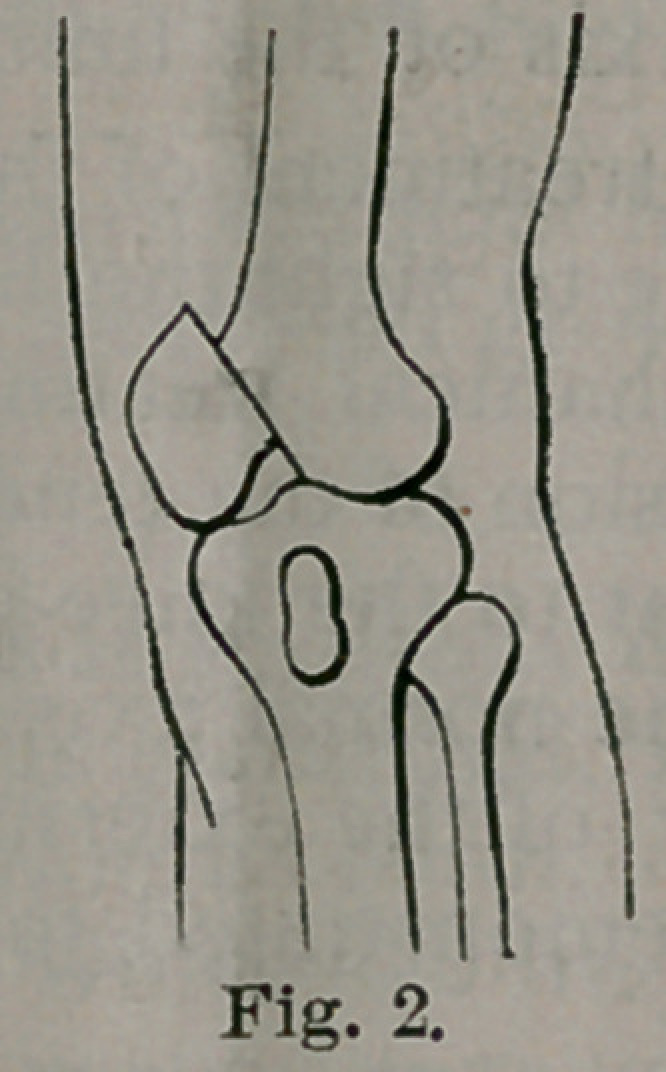


**Fig. 3. f3:**
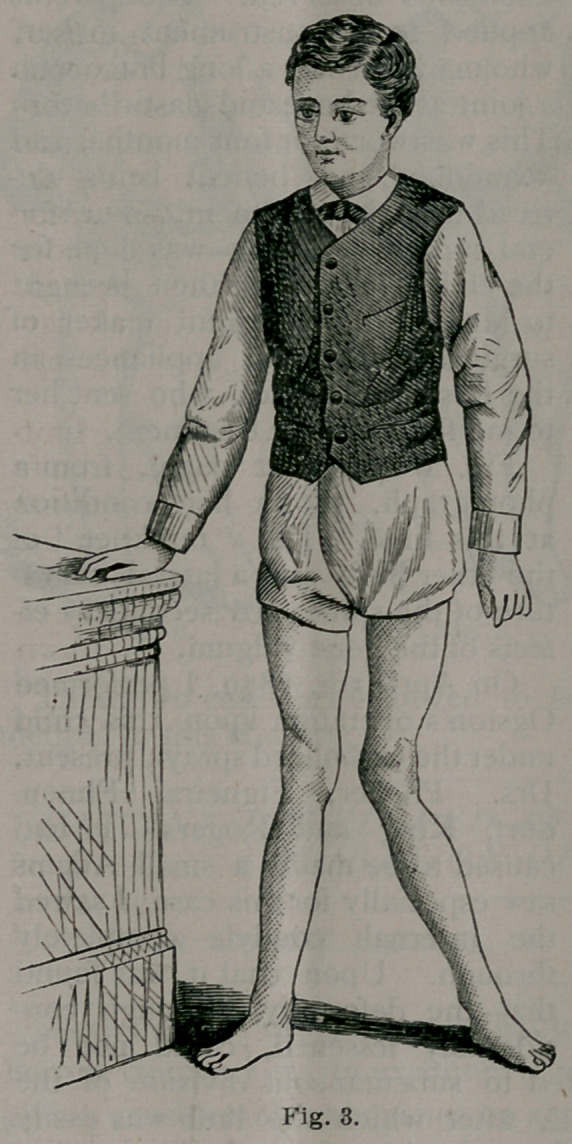


**Fig. 4. f4:**
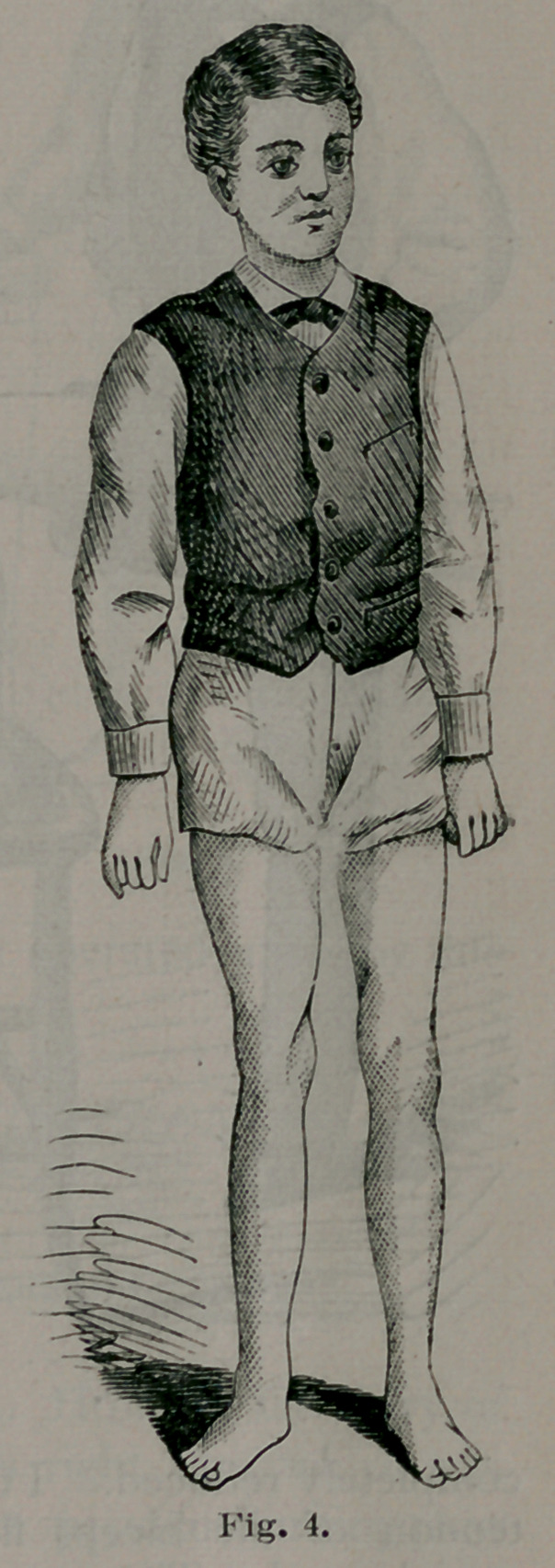


**Fig. 5. f5:**
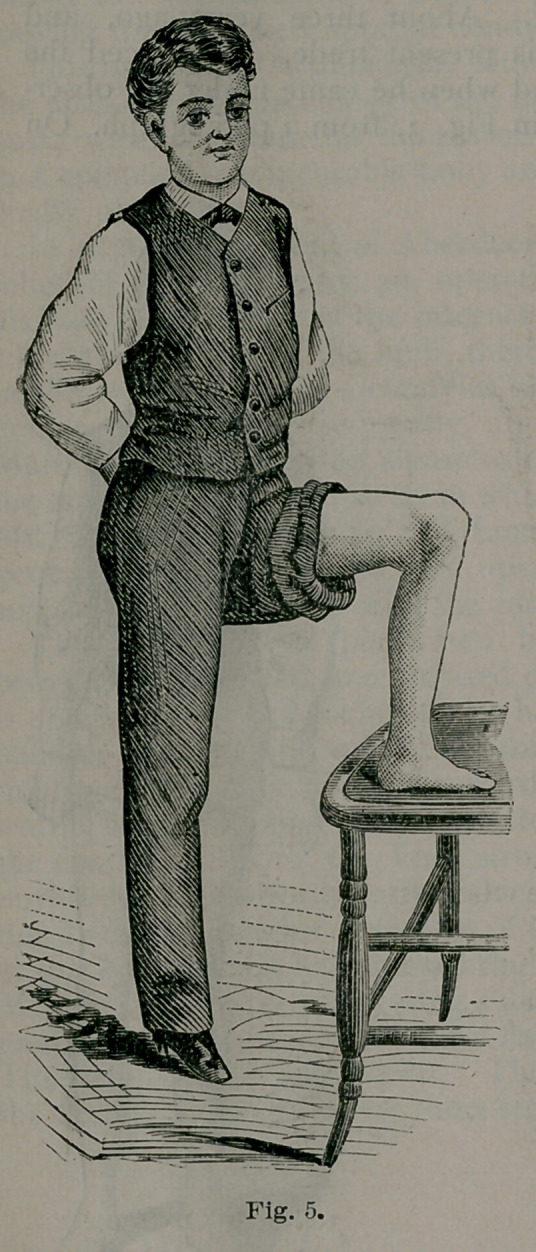


**Fig. 6. f6:**
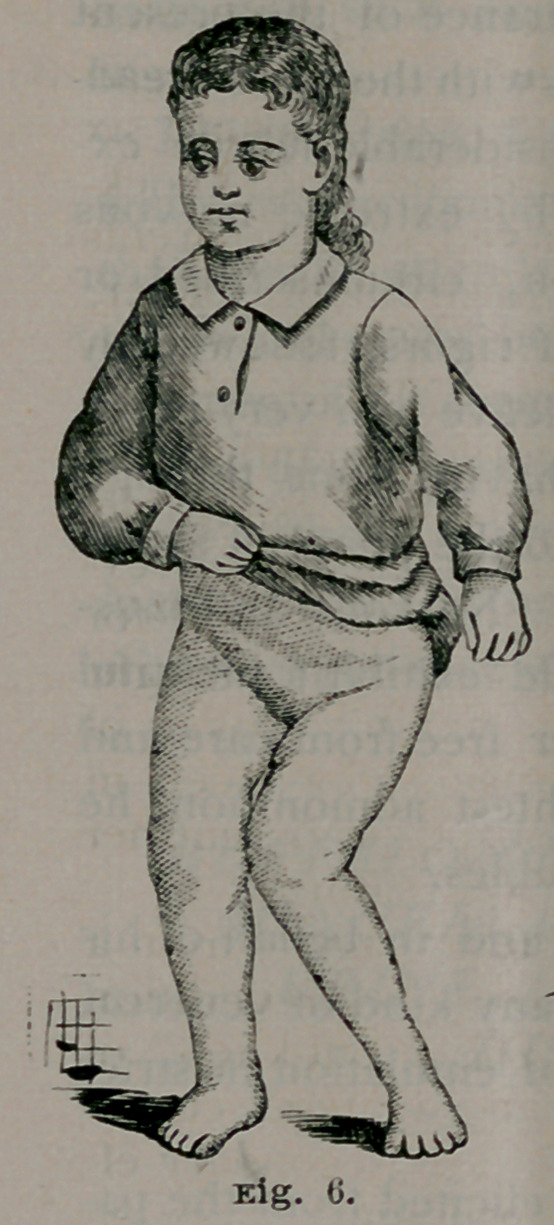


**Fig. 7. f7:**
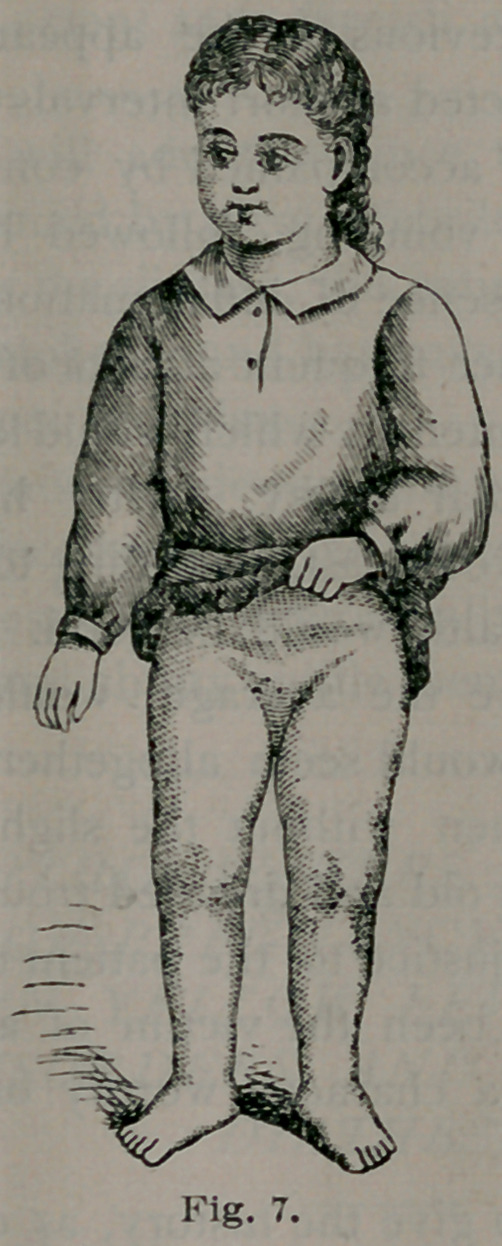


**Fig. 8. f8:**